# Efficient Feature Learning Model of Motor Imagery EEG Signals with L1-Norm and Weighted Fusion

**DOI:** 10.3390/bios14050211

**Published:** 2024-04-23

**Authors:** Xiangzeng Kong, Cailin Wu, Shimiao Chen, Tao Wu, Junfeng Han

**Affiliations:** 1 College of Mechanical and Electrical Engineering, Fujian Agriculture and Forestry University, Fuzhou 350108, China; xzkong@fafu.edu.cn; 2 School of Future Technology, Fujian Agriculture and Forestry University, Fuzhou 350002, China; 52362047015@fafu.edu.cn (C.W.); 5226239003@fafu.edu.cn (S.C.)

**Keywords:** electroencephalogram, feature selection, mutual information, penalty term, weighted fusion

## Abstract

Brain–computer interface (BCI) for motor imagery is an advanced technology used in the field of medical rehabilitation. However, due to the poor accuracy of electroencephalogram feature classification, BCI systems often misrecognize user commands. Although many state-of-the-art feature selection methods aim to enhance classification accuracy, they usually overlook the interrelationships between individual features, indirectly impacting the accuracy of feature classification. To overcome this issue, we propose an adaptive feature learning model that employs a Riemannian geometric approach to generate a feature matrix from electroencephalogram signals, serving as the model’s input. By integrating the enhanced adaptive L1 penalty and weighted fusion penalty into the sparse learning model, we select the most informative features from the matrix. Specifically, we measure the importance of features using mutual information and introduce an adaptive weight construction strategy to penalize regression coefficients corresponding to each variable adaptively. Moreover, the weighted fusion penalty balances weight differences among correlated variables, reducing the model’s overreliance on specific variables and enhancing accuracy. The performance of the proposed method was validated on BCI Competition IV datasets IIa and IIb using the support vector machine. Experimental results demonstrate the effectiveness and superiority of the proposed model compared to the existing models.

## 1. Introduction

Motor imagery (MI) involves the subject’s mental imagery of body movements without actual physical movement. The electroencephalogram (EEG) signals generated by MI exhibit special characteristics linked to the conscious activation of brain areas, and these signals can be extracted using signal processing techniques [1]. Brain-computer interface (BCI) is a highly significant class of interaction strategy between the brain and machines, as it can collect, amplify, and process neural signals from the brain. The most important core of the BCI system is able to decode the user’s subjective action intention from the detected EEG signals, and then the EEG can be translated into a control signal of external devices by an appropriate pattern recognition algorithm, enabling direct communication between humans and machines [2]. In recent years, BCI has emerged as a promising technology with significant contributions towards medical applications, such as stroke rehabilitation [3], wheelchair control [4], and prosthetic limb control [5]. However, the current BCI system frequently identifies erroneous user commands which leads to low accuracy and message transfer rates. Therefore, it is highly desirable to propose a technique that can improve the accuracy of decoded MI-EEG signals for the BCI system [6].

Generally, the decoding process of MI-EEG signals primarily involves feature extraction and classification. The challenge of feature extraction, namely extracting various signal features, is a fundamental issue that garners significant attention in the literature [7,8]. Various approaches have been proposed, for instance, Wavelet Transform [9], Short-Time Fourier Transform [10], spectrogram and autoregressive [11], and Common Space Pattern (CSP) [12]. Among them, the CSP algorithm is considered to be one of the most effective feature extraction algorithms for the MI-EEG [13]. However, the calculation of the covariance matrix in the CSP algorithm is prone to interference from noisy or abnormal samples. Original EEG data are high-dimensional with a low signal-to-noise ratio; therefore, a large number of high-dimensional features will be generated by the CSP algorithm [14]. On the other hand, Riemannian geometry (RG), one of the machine learning techniques, has been utilized for classification since it can better capture the correlation and internal structure of EEG signals and perform well in handling high-dimensional data [15,16]. Therefore, the first motivation of this paper is to explore a new computational algorithm that can simplify the signal processing and improve the signal-to-noise ratio of the MI-EEG via the Riemannian manifold.

As feature extraction will generate large feature sets, feature selection is crucial in the analysis of EEG signals, particularly when addressing the challenges posed by big data characteristics such as velocity and variety [17]. Classically, based on the relationships among classification models, feature selection methods can be categorized as the filter method, wrapper method, and embedded method [18]. The filter method in EEG signal processing involves selecting a subset of features based on their statistical properties or relationships with the target variable, such as Correlation-Based Feature Selection (CFS) [19] and the *t*-test algorithm [20]. The CFS algorithm calculates a subset of the feature by following the initial hypothesis to identify features that are highly correlated with the target variable. Even though it is computationally efficient and easy to implement in the feature selection process for EEG signals, CFS ignores feature interactions that might not capture non-linear relationships. By applying statistical tests to identify features of the EEG signals, the *t*-test method provides information about the statistical significance of differences in means between groups. The mutual information algorithm selects features with high information content by measuring the information gain or mutual information between each feature and the target variable [21]. It is worth noting that the methods mentioned above primarily focus on individual feature selection, potentially missing the complex relationships among features. By the global search in EEG signal processing, wrapper methods for feature selection involve evaluating subsets of features based on the performance of a specific machine learning model, such as Recursive Feature Elimination (RFE) [22] and Genetic Algorithms (GAs) [23]. RFE and GAs not only consider the feature interactions but the least important features are removed until the desired number of features is reached. However, wrapper methods are computationally intensive for high-dimensional data.

Embedded methods integrate feature selection into the model training process, selecting important features by optimizing the objective function [24]. These methods are computationally less intensive than wrapper methods and exhibit superior performance compared to filter methods [25]. The regularization model is a vital embedded method capable of shrinking features on a continuous scale and achieving automatic feature selection. One representative individual regularization model is the least absolute shrinkage and selection operator (LASSO), which minimizes the combination of loss functions and L1 regularization terms to eliminate the weight of irrelevant features [26]. It selects one feature from a group of highly correlated features, which can help simplify models and improve generalization performance by effectively providing a form of dimensionality reduction. However, LASSO assumes a linear relationship between the features and the target variable. While LASSO demonstrates effectiveness in feature selection within EEG signal processing, it may exclude potentially vital features that hold significance in the overall analysis. Therefore, another motivation of this paper is to consider the interaction features of each feature for EEG feature selection, with a particular focus on capturing the complex relationships among the features in the high-dimensional data of the selected features.

In this paper, we present a novel machine learning framework designed to address the challenges associated with decoding the MI-EEG signals. Our framework is built upon RG and incorporates variants of adaptive LASSO. The Riemannian distance serves as a metric to assess the redundancy of features in the detected EEG signals. Relevant features are then extracted to reduce the dimensionality of the feature data. Then, to enhance the classification accuracy of the system, we integrate mutual information-based and pairwise-fused (MIPF) LASSO to selectively identify features with interactions and redundancies. Finally, support vector machine (SVM) classification is employed to process high-dimensional data and capture non-linear relationships within feature sets. The primary contributions of this paper can be summarized as follows:

1. A new adaptive MIPF-LASSO model is developed for feature selection in MI-EEG signals classification. We improve the original LASSO model by using two regularization terms: adaptive L1 and weight fusion. The former aims to adaptively assign different weights to different feature coefficients, and the latter aims to group variables by using relevant information data.
2. A new adaptive weight construction strategy is proposed that can adaptively penalize the regression coefficients corresponding to each variable by measuring the importance of each feature through mutual information.
3. A new pattern recognition framework for MI-EEG signals is proposed. Experimental results show that extracting the most informative features is expected to improve the accuracy and feasibility of EEG analysis and provide a powerful tool for neuroscience research and brain imaging applications.

The remainder of this paper is structured as follows: Section 2 provides a brief overview of related studies on feature selection methods. Section 3 outlines the proposed method. Section 4 presents comparative experimental results and discussions. Finally, Section 5 concludes the paper and outlines directions for future research.

## 2. Related Work

In recent years, considering the challenges posed by big data characteristics like velocity and variety, feature selection is crucial in the analysis of EEG signals. LASSO can perform automatic feature selection from a group of highly correlated features by introducing an L1 regularization term, effectively providing an expression of the dimensionality reduction [27]. However, in the case of highly correlated features, LASSO may exhibit instability, manifested as inconsistency in feature selection. To overcome this issue, grouped LASSO models play a crucial role in maintaining the spatial or functional consistency of the selected features. This is achieved by considering the similar functionality of features and applying common penalties to the entire group of features [28]. However, the effectiveness and the performance of these models may be sensitive to the accurate definition of groups [29]. Determining the optimal grouping structure can be a challenge to the choice of groups. As these models use the same tuning parameters for all regression coefficients, the resulting estimators may have significant biases.

To ameliorate this shortcoming, by using constructed adaptive weights, the adaptive LASSO is proposed to adaptively select the relative feature, in which adaptive weights are employed to penalize different coefficients in the L1-penalty [30]. So, adaptive LASSO has the ability to adapt the strength of the regularization to different features, particularly useful in dealing with EEG signals. Even though the oracle properties of identifying the correct subset model are preserved in adaptive LASSO, the weights established by these methods are constructed based on the initial consistent estimator, which is sensitive to noise or abnormal signal values in the EEG dataset. To modify adaptive LASSO, numerous weight construction techniques have been proposed to select features with complex relationships. Since the EEG signals often involves non-linear interactions, these weights are highly dependent on the actual values of the original data. Therefore, many feature selection methods based on information theory have been developed to characterize the complex dynamic behaviors of EEG signals [31,32]. Mutual information, a typical method of information theory, is used to analyze relationships among different EEG channels or EEG signals and external stimuli [33]. In summary, mutual information is a powerful tool for capturing dependencies and interactions in EEG signals, especially in cases involving non-linear relationships [34]. While candidate feature relevancy is considered to be equivalent to selected feature relevancy in mutual information, some less relevant features may be misinterpreted as salient features. To overcome these issues, the fusion LASSO enhances the trade-off between the relevancy of each individual feature [35]. However, due to the structured penalties, the fusion LASSO cost is computationally high when dealing with EEG dataset. Since fusion LASSO involves tuning parameters, it is important to note that the method is sensitive to noise. Therefore, the performance of the method may be affected by the choice of tuning parameters. In [36], the segmentation-denoising network is proposed to improve the sensitivity and specificity of EEG signals.

Most of the aforementioned studies have often overlooked the potential of correlated features in improving EEG classification performance. Therefore, this paper combines the advantages of the adaptive LASSO algorithm and proposes a weight construction strategy that utilizes mutual information to measure feature relevance, aiming to improve the classification performance of EEG signals.

## 3. Material and Methods

**Figure 1:**
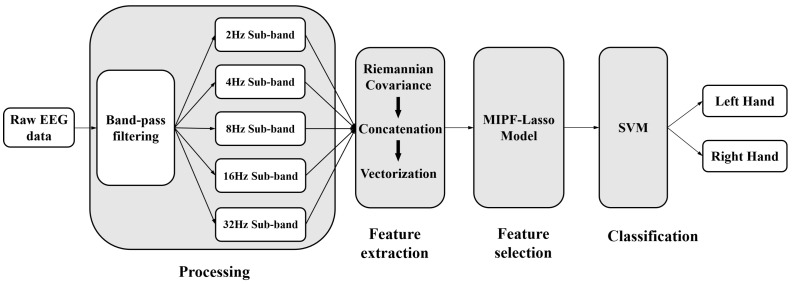
The overview of our proposed framework for motor imagery classification.

In this study, we propose an innovative framework for EEG signal classification. The algorithmic overview of this framework is illustrated in Figure 1. Specifically, we divide the original EEG signals into different time windows. Within each time window, we perform spectral analysis to obtain significant features of EEG signals in different frequency ranges. Secondly, we use the feature extraction algorithm of RG, which directly manipulates the covariance matrix of EEG signals in space. This step allows us to obtain structural information about the synergistic activity between brain regions. Then, we introduce the MIPF-LASSO model proposed in this paper, which provides an efficient and accurate feature representation for classification tasks by learning and selecting informative features. Finally, the obtained features are input into an SVM classifier to obtain the classification results.

### 3.1. EEG Datasets

In this study, we use two publicly available datasets to evaluate the effectiveness of our proposed method.

**Figure 2:**
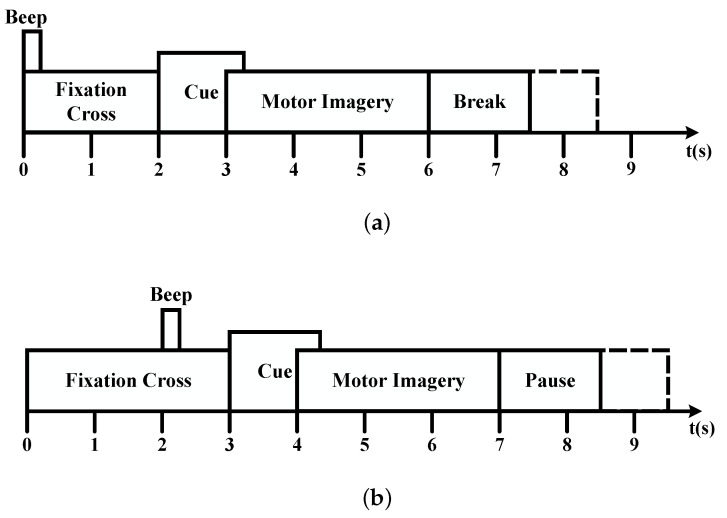
Timing paradigm of each trial. (**a**) BCI competition IV dataset IIa. (**b**) BCI competition IV dataset IIb.

BCI Competition IV Dataset IIa: The dataset recorded 22 EEG signal channels from nine subjects at a sampling rate of 250 Hz. Each participant in the trial was given instructions to perform four different kinds of MI tasks using visual cues: left hand, right hand, foot, and tongue. Two sets of MI task data were recorded by each individual. One set was used to train the model, and the other to assess the model’s effectiveness. Participants completed six different sets of MI tasks in each phase; each set was repeated twelve times, for a total of 144 experimental trials in each phase. The timing scheme of each trial is shown in Figure 2a. In this paper, the time interval of single EEG data is limited to 2.5∼6 s. The details of this dataset are available in [37].

BCI Competition IV Dataset IIb: The dataset recorded 3 EEG signal channels (C3, Cz, and C4) from nine subjects at a sampling rate of 250 Hz. Each participant in the trial was given instructions to perform two different kinds of MI tasks using visual cues: left hand and right hand. Each data collection session consisted of 120 trials of data. The subject was indicated by a visual cue to perform the MI task for 4.5 s. The timing scheme for each trial is shown in Figure 2b. The details of this dataset are available in [37].

### 3.2. Preprocessing

Given that the number of subjects obtaining EEG data collection in reality is relatively small, this is not conducive to learner training [38] due to the high temporal resolution nature of the signals, i.e., the large amount of information that can be provided in a short period of time. We can use effective data enhancement methods to increase the diversity and quantity of training data. First, we divide the data from 0.5 s before the start of the MI task to the end of the task into time windows and divided them into six different windows. The time segments ($T_{1}$ to $T_{6}$) that contain the temporal information of MI classification are successively 2.5–4.5, 4–6, 2.5–6, 2.5–3.5, 3–4, and 4–5. In this study, we choose the largest time window ($T_{3}$) for the experiment.

MI can cause event-related desynchronization (ERD) and event-related synchronization (ERS), i.e., power changes in specific frequency bands of EEG signals, specifically sensory-motor rhythms mu (8–13 Hz) and beta (15–30 Hz). As a result, the band-pass filter of 8–30 Hz is usually used to filter the MI signals [39]. However, since the frequency response of MI is subject-specific, it is difficult to separate the most discriminative features only in the mu and beta rhythms [29,40]. Therefore, it is crucial to extend the frequency band of the EEG signals and divide it into multi-scale spectral parts before the feature extraction stage. Therefore, we choose 5 different types of frequency bandwidth (2 Hz, 4 Hz, 8 Hz, 16 Hz, and 32 Hz) for multi-scale spectral segmentation and employed a second-order Butterworth band-pass filter, which is an infinite impulse response (IIR) filter with frequency bands ranging from 4 to 40 Hz to perform multi-scale spectral segmentation, further helping us enhance or select the signal components in a specific frequency range.

### 3.3. Feature Extraction

The decomposition of the EEG signals into frequency sub-bands after preprocessing results in increased dimensionality [41]. To address this issue, it is necessary to extract effective features for use in EEG recognition. In contrast to the classic CSP algorithm series, which attempts to find the best spatial filter only for binary classification, the use of RG broadens the horizons of MI-EEG decoding beyond spatial filters. On the one hand, the traditional decoding methods assume that the EEG signals is located in high-dimensional Euclidean space and then directly process the signal based on the Euclidean distance. However, since each dimension of EEG corresponds to a different channel, in addition to the presence of phase information, the representation of EEG in multidimensional space exhibits non-Euclidean properties. RG provides a framework for exploring non-Euclidean spaces, which aids in precisely describing the internal relationships of high-dimensional EEG data [42]. On the other hand, RG can explain the geometric properties on matrix manifolds. It is more appropriate to deal with MI-EEG covariance matrices, which can directly process the covariance features of EEG signals, thereby improving the accuracy of EEG decoding [15].

In this study, we apply the Riemann geometry technique to reduce dimensionality and improve the discriminability of the datasets between different MI classes. Specifically, we compute the covariance matrix for each sub-band to obtain a symmetric positive definite matrix, and every real symmetric positive definite matrix corresponds to a point on a Riemannian manifold. We employ logarithmic mapping for the vector projection from a sub-manifold to its corresponding tangent space and utilize exponential mapping to project points back onto the sub-manifold from the tangent space. Given the eigenvalues of the MI-EEG covariance matrix, we use the geodesic distance to quantify the differences between the covariance matrices. For the different classes, we consider the measures between numerous covariance matrices from the mean perspective. By using the Riemann mean matrix based on MI-EEG signals, we generate a new feature matrix which is then vectorized. These steps constitute the Riemann covariance method, which realizes the robust feature extraction of MI-EEG by capturing the dynamic features of the MI-EEG signals.

### 3.4. Adaptive LASSO with Mutual Information and Weighted Fusion

After obtaining feature representations on a Riemannian manifold, the accuracy of the decoding may potentially be improved. However, this will result in the generation of a huge number of features. So, we propose an adaptive feature selection model. Based on the LASSO algorithm, the regression coefficients corresponding to each variable are adaptively penalized by introducing weighting coefficients constructed based on mutual information. At the same time, it enables the automatic grouping effects of regression coefficients. In this way, the model can select the features related to the target variables more accurately, reduce the selection bias, and maintain the consistency of model selection.

#### 3.4.1. Adaptive Weight Strategy

In this section, we propose a new adaptive weight construction strategy, in which the key point is to measure the importance of each feature to the target task based on mutual information. To ensure completeness, we give some basic concepts of information theory. In this theory, due to entropy’s ability to quantify the uncertainty of random variables and effectively measure the amount of information shared between random variables, it has been widely used in many fields [43].

Let $X={\left[x_{1},x_{2},\cdots ,x_{n}\right]}^{T}$ be the set of discrete random variables, and its uncertainty can be measured by entropy H(X), which is represented as:

(1) $$\;H\left(X\right)=-\sum_{x\in X}p\left(x\right)\log p(x)$$

where p(x) is the probability distribution of each *x*. The greater the entropy H(X) of random variable *X* is, the more information it contains. When certain variables are known and others are not, the remaining uncertainty is measured by the conditional entropy. The conditional entropy of two random variables *X* and *Y* is defined as follows:

(2) $$\;H\left(X|Y\right)=-\sum_{y\epsilon Y}p\left(y\right)\sum_{x\epsilon X}p\left(x|y\right)\log p(x|y)$$

where p(y) and p(x∣y) are the probability of *y* and probability of *x* given *y*, respectively. Here, the conditional entropy H(X∣Y) represents the degree of uncertainty of *X* given *Y*.

Mutual information is a metric of interdependence between random variables. Thus, it provides a way to assess the relevance of a subset of features. In the following, we introduce mutual information. Let $I_{j}$ represent the individual importance of the *j*-th feature, which can be expressed as:

(3) $$\;I_{j}=I\left(X_{\left(j\right)};Y\right)=\sum_{x_{j}\in X}\sum_{y\in Y}p\left(x_{j},y\right)\log\frac{p(x_{j},y)}{p\left(x_{j}\right)p\left(y\right)}$$

where $p(x_{j},y)$ denotes the joint probability density function of $x_{j}$ and *y*. Here, $I(X_{\left(j\right)};Y)$ can indicate the correlation between feature $x_{\left(j\right)}$ and class *y*. From this definition, if $I(x_{\left(m\right)},y)$ exceeds $I(x_{\left(n\right)},y)$, it means that the *m*-th feature $x_{\left(m\right)}$ contains more information about class *y* than the *n*-th feature $x_{\left(n\right)}$. Therefore, $I_{j}$ can be regarded as a specific index to measure the significance of features, that is, the higher the value of $I_{j}$, the more significant the feature $x_{\left(j\right)}$. In particular, if the feature $x_{\left(j\right)}$ does not provide any substantial information for the class label, then $I_{j}=0$.

In order to impose different penalties on each feature according to its importance for the classification, we construct the weight coefficient of the *j*-th feature based on $I_{j}$, which can be defined as:

(4) $$\alpha_{j}=e^{-rI_{j}}$$

where *r* is the trade-off parameter. In this study, the kernel transformation is applied to the construction of the weighting coefficients. Through repeated experimental validation, we observe that the performance of the model reaches the optimal level when *r* = 5. Therefore, we decide to set *r* to 5 during further model tuning.

After computing the weight coefficient corresponding to the *j*-th (j=1,⋯,p) feature, we describe the weights matrix as:

(5) $$\;A=diag(\alpha_{1},\cdots ,\alpha_{p})$$

#### 3.4.2. The MIPF-LASSO Model

We propose a feature learning model based on adaptive weights and paired fusion LASSO. For extracting the most distinctive features, we choose the features extracted from the RG as the input feature matrix $X=\left[x_{1},x_{2},\cdots ,x_{p}\right]\in R^{n\times p}$, which consist of *n* samples with *p*-dimensional features. In this model, the features corresponding to the non-zero elements of these sparse $\hat{\beta }$ in the input feature matrix X are selected as candidate features in the classification task.

Let $y={\left(y_{1},\cdots ,y_{n}\right)}^{T}$ be the response vector. Then, our model is formulated as:

(6) $$\hat{\beta }\left(\lambda_{1},\lambda_{2}\right)=\underset{\beta }{argmin}{∥y-X\beta ∥}^{2}+\lambda_{1}\sum_{j=1}^{p}\alpha_{j}\left|\beta_{j}\right|+\lambda_{2}J\left(\beta \right)$$

where $\beta \in R^{p}$ is the vector of regression parameters. $\lambda_{1}$ and $\lambda_{2}$ are tuning parameters that are used to regulate the degree of different penalties. The model has two penalty terms. The first term is a new adaptive L1 penalization, and the second one is pairwise-fused penalization, which are described below, respectively:

(1) For adaptive L1 penalization, we propose a new weight coefficient construction strategy, where $\alpha_{j}$ can be calculated as Equation (4), $\lambda_{1}$ controls the degree of sparsity of the $\hat{\beta }$, and a higher $\lambda_{1}$ results in a rarer $\hat{\beta }$, and thus a smaller number of features can be extracted.
(2) For pairwise-fused penalization, it penalizes the pairwise difference of the estimated coefficients by using the correlate-driven weights with the help of relevant information in the data. $\lambda_{2}$ focuses on correlations between spectral features, and strongly correlated features are retained or removed at the same time. This allows our model to be very suitable for handling the data with strongly correlated, multi-collinear predictors.
  Let $\rho_{ij}=x_{i}^{T}x_{j}$ denotes the sample correlation between the predictors $x_{i}$ and $x_{j}$. The weighted fusion penalty J(β) is defined as:
  (7) $$J\left(\beta \right)=\sum_{j=1}^{p-1}\sum_{i>j}\left\{\frac{{\left(\beta_{i}-\beta_{j}\right)}^{2}}{1-\rho_{ij}}+\frac{{\left(\beta_{i}+\beta_{j}\right)}^{2}}{1+\rho_{ij}}\right\}$$
  According to [44], the function (7) possesses the fusion weight characteristics. By definition, we can see that for the strong positive correlation ($\rho_{ij}\approx 1$), the first term dominates and $\hat{\beta_{i}}$ will be close to $\hat{\beta_{j}}$. The method explicitly utilizes the correlation between predictors in the weighted fusion penalty term. The coefficients corresponding to pairs of covariates are weighted according to their marginal correlations. From an application point of view, edge weights can be used to measure the similarity between two vertices. By considering edge weights, the weighted fusion method can select highly correlated variables as a group. Even though the true coefficients of this group of variables may not be equal, the use of edge weights allows the model to more flexibly deal with these highly correlated variables and make predictions as a whole, achieving a group effect, which improves the model’s adaptability to the group of highly correlated variables, thus improving the accuracy of the prediction.

Consider an optimization algorithm when λ=0. The solution of our proposed algorithm can be transformed into:

(8) $${\hat{\beta }}^{RF}\left(\lambda_{2}\right)={\left(X^{T}X+\lambda_{2}W\right)}^{-1}X^{T}y$$

where

$$W=\left(\begin{array}{cccc} \sum_{j\neq 1}\frac{1}{1-\rho_{1j}^{2}} & -\frac{\rho_{12}}{1-\rho_{12}^{2}} & \ldots & -\frac{\rho_{1p}}{1-\rho_{1p}^{2}}\\ -\frac{\rho_{12}}{1-\rho_{12}^{2}} & \sum_{j\neq 2}\frac{1}{1-\rho_{2j}^{2}} & \ldots & \vdots \\ \vdots & \vdots & \ddots & -\frac{\rho_{p-1,p}}{1-\rho_{p-1,p}^{2}}\\ -\frac{\rho_{1p}}{1-\rho_{1p}^{2}} & \ldots & -\frac{\rho_{p-1,p}}{1-\rho_{p-1,p}^{2}} & \sum_{j\neq p}\frac{1}{1-\rho_{pj}^{2}} \end{array}\right)$$

The matrix W is symmetric and semi-definite, and ${\hat{\beta }}^{RF}$ is defined as the solution to the ridge fusion estimation. When the number of features (p) is greater than the number of samples (n), the covariance matrix $X^{T}X$ may become a singular matrix. Ridge regression increases the rank of the covariance matrix by adding a constant to it, and the ridge fusion method increases the rank of $X^{T}X$ by adding a matrix W, i.e., by adjusting the magnitude of the correlation coefficient, which also solves the correlation-based p>n problem.

We have improved the MIPF-LASSO algorithm to solve the penalty least squares problem. Our main idea is to introduce data augmentation so that the weighted fusion problem can be transformed into a cable problem. A nice characteristic of the function (7) is that it may be expressed as a simple quadratic form, which allows to provide the resulting estimator in closed form:

(9) $$J\left(\beta \right)=\beta^{T}W\beta$$

We can rephrase the optimization problem as:

(10) $$\hat{\beta }\left(\lambda_{1},\lambda_{2}\right)=\underset{\beta }{argmin}\left\{{∥y-X\beta ∥}^{2}+\lambda_{1}\sum_{j=1}^{p}\alpha_{j}\left|\beta_{j}\right|+\lambda_{2}\beta^{T}W\beta \right\}$$

Because W is positive semi-definite, we can use Cholesky decomposition to obtain $W=R^{T}R$, where R is the upper triangle matrix. Let

(11) y^(n+p)=y0,X^(n+p)×p=Xλ2R

The formula (10) can further be rewritten as:

(12) $$\hat{\beta }\left(\lambda_{1},\lambda_{1}\right)=\underset{\beta }{argmin}\left\{{∥\hat{y}-\hat{X}\beta ∥}^{2}+\lambda_{1}\sum_{j=1}^{p}\alpha_{j}\left|\beta_{j}\right|\right\}$$

Define $X^{*}=\hat{X}/\alpha$, then we have

(13) $$\hat{\beta^{*}}\left(\lambda_{1},\lambda_{1}\right)=\underset{\beta }{argmin}\left\{{∥y^{*}-X^{*}\beta^{*}∥}^{2}+\lambda_{1}{∥\beta^{*}∥}_{1}\right\}$$

which implies that the computation of the MIPF-LASSO estimator can be handled by available algorithms for LASSO, such as Least Angle Regression (LARS) or coordinate descent (CD) algorithms [45]. Among them, the coordinate descent method only updates the coefficient of one feature in each iteration, while the coefficients of other features remain unchanged, which makes the algorithm run faster. Through repeated iterations, one can eventually find the parameters that minimize the objective function. Therefore, our model adopts the coordinate descent algorithm, which can be seen in Algorithm 1. Given that the objective function of problem (13) is divided into two parts, the first part being a differentiable convex function and the second part being a convex function, we can ensure the convergence of Algorithm 1 according to Theorem 1. Assuming that *n* is the number of samples and *p* is the number of features, the computational complexity of Algorithm 1 is O(np).

**Theorem** **1.** *Reference* [46] *assumes that the level set $X^{0}=\left\{x:f\left(x\right)\leq f\left(x^{0}\right)\right\}$ is compact and that the function f is continuous on $X^{0}$. Then, the sequence ${\left\{x^{r}=\left(x_{1}^{r},\cdots ,x_{N}^{r}\right)\right\}}_{r=0,1,\cdots }$ generated by the coordinate descent method using the essentially cyclic rule is defined and bounded. In addition, if for every $i,k\in \left\{1,\cdots ,N\right\}$, the function $f(x_{1},\cdots ,x_{N})$ in $\left(x_{k},x_{i}\right)$ is pseudoconvex and f at every $x\in X^{0}$ is regular, then every cluster point of $\left\{x^{r}\right\}$ is a stationary point of f.*

**Remark** **1.** When the number of features p is much larger than the number of samples n, the traditional LASSO method may encounter limitations in variable selection because in this case, the number of parameters in the model far exceeds the number of available data points, which may lead to over-fitting problems. Typically, the coordinate descent algorithm is used to speed up the optimization process in LASSO solving, but it does not solve the challenge of the number of features being greater than the number of samples. To overcome this problem, an improvement is proposed by applying the coordinate descent algorithm to augmented data ($y^{*}$, $X^{*}$). This improvement not only extends the applicability of LASSO to effectively deal with situations where the number of features is larger than the number of samples but also resembles the traditional LASSO method in terms of variable selection. Therefore, by using augmented data in the coordinate descent algorithm, the limitation of traditional LASSO in the p>n dilemma is successfully overcome.

**Algorithm 1:** Algorithm for MIPF-LASSO.

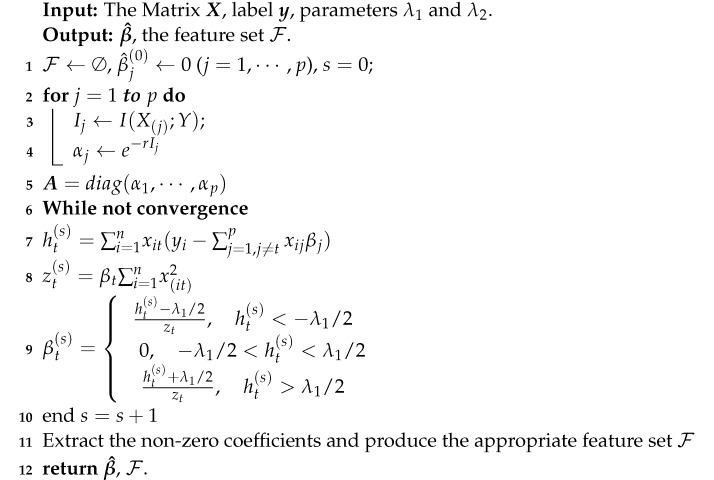



### 3.5. EEG Classification

After obtaining the EEG features learned from the MIPF-LASSO model, a variety of classical classifiers are available for detection tasks, such as random forest, linear discriminant analysis and SVM. SVM is a typical classification method that has been widely used in MI-BCI, which maps the original feature to the higher dimensional space by kernel function and deals with the linear indivisible problem in the original feature space, making it more flexible in the classification of high-dimensional data. In addition, SVM pursues a hyperplane that maximizes spacing, making it more robust to small sample datasets and more sensitive to core samples, helping to resist the effects of noise. Considering that the features selected by the proposed model still involve high-dimensional feature spaces and small sample datasets, to effectively deal with this challenge, this paper adopts SVM as the classifier. Kernel functions typically employed in SVM include linear, polynomial, radial basis function (RBF), and sigmoid functions. The RBF kernel function is a typical non-linear kernel function that can map data to a high-dimensional feature space while also having outstanding generalization and adaptability, making it particularly ideal for dealing with non-linear separable classification issues. As a result, we choose to train SVM with RBF kernel functions to accurately identify the MI tasks.

## 4. Results and Discussion

### 4.1. Experimental Setting

To assess the effectiveness of our proposed framework, we use two widely accepted evaluation metrics, including accuracy and G-mean. Accuracy is the ratio of the number of correctly classified samples to the total number of samples, and it is one of the most commonly used indicators in classification performance evaluation. However, in the case of dealing with unbalanced datasets, accuracy may not provide sufficient information. The F1 score is a harmonic average of the accuracy rate and sensitivity, which takes into account the accuracy and sensitivity performance of the model and is particularly suitable for balancing different aspects of performance. G-mean is a complete assessment statistic that incorporates the classifier’s recall and accuracy rates and can comprehensively evaluate the classifier’s performance. When working with unbalanced datasets, this index is extremely useful for analyzing the categorization effect.

The following is the mathematical formula for these indicators:

(14) $$\;Accuracy=(\frac{TP+TN}{TP+FP+TN+FN})$$

(15) $$\;F1-score=\frac{2TP}{2TP+FP+FN}$$

(16) $$\;G-mean=\sqrt{\frac{TP}{TP+FN}\times \frac{TN}{TN+FP}}$$

where TP (true positive) and TN (true negative) represent the count of accurately identified true abnormal and true normal instances, respectively. FP (false positive) characterizes instances that are incorrectly classified as abnormal, while FN (false negative) denotes instances erroneously labeled as normal.

### 4.2. Comparison with Other State-of-the-Art Studies

To verify the efficacy of the proposed MIPF-LASSO method, we implement a comprehensive series of experimental comparisons. The experiments involve pitting our method against other cutting-edge methodologies through rigorous evaluations of identical datasets. This meticulous and extensive examination was undertaken to validate and demonstrate the superior performance of the MIPF-LASSO method in comparison to the state-of-the-art alternatives. The dataset used is the same as that of the work of Autthasan P. et al. [47]. These state-of-the-art methods include the following:

- FBCSP+SVM [48]: The FBCSP technique was devised to apply the original CSP algorithm to each sub-band of EEG signals, thereby extracting discernible EEG features from multiple frequency bands, which can maximize the variance of different EEG signals. Subsequently, the SVM is trained to classify the extracted features. Finally, the SVM classifier with optimal parameters is used for testing.
- FBCSP+LDA [49]: Reference [49] evaluates the performance of five popular MI-BCI pipelines, allowing BCI researchers to select the best BCI pipeline for their purpose. We choose one of the FBCSP+LDA algorithms to simulate on datasets IIa and IIb. Specifically, the FBCSP algorithm divides the preprocessed EEG signals into a series of band-pass filtered signals, then applies the CSP algorithm and LDA classifier to each band individually, followed by score fusion and classification.
- Spectral–Spatial CNN [50]: The spectral–spatial relationship CNN is a novel spectral–spatial feature representation framework based on CNNs from a large-scale MI-EEG database. The framework learns the spectral–spatial input, extracting discriminative properties from the different frequency bands of the EEG signals. It achieves cutting-edge performance in subject-independent MI decoding.
- Deep ConvNet [51]: As a deep learning model, Deep Convolutional Neural Networks are designed based on the classical CNN architecture and have been shown to be effective for decoding MI-EEG signals. In our experiments, Deep ConvNet was optimally configured as recommended in [51].
- EEGNet [52]: EEGNet is a lightweight CNN framework for classification in different BCI paradigms. There are different versions of EEGNet depending on the network parameters. In our experiments, we chose EEGNet-8,2 for a fair comparison and followed the description in the original publication.
- MIN2Net [47]: MIN2Net develops by combining an autoencoder, deep metric learning, and a supervised classifier, which simultaneously learns to compress, differentiate embedded EEG, and classify EEG. MIN2Net performs excellently in terms of subject independence.

The comparisons between the proposed method and alternative approaches, as presented in Table 1 and Table 2 using BCI Competition IV datasets IIa and IIb, emphasize the efficacy of our proposed MIPF-LASSO method. Within the confines of Table 1, a comprehensive comparison unfolds to indicate the exceptional performance of MIPF-LASSO alongside other methodologies, revealing a clear advantage in classification accuracy by a striking margin of no less than 7.39% when compared with state-of-the-art methodologies. The F1-Score showcases an impressive lead of at least 8.2% in favor of MIPF-Lasso. Furthermore, the G-mean, a robust metric of performance, unfurls an extraordinary advantage of a minimum of 7.07%, firmly placing MIPF-LASSO in a position of remarkable superiority.

Expanding our analysis to BCI Competition IV dataset IIb, a comparison with the six alternative methods not only reveals the excellence of our proposed methodology but also demonstrates a substantial enhancement in both classification accuracy and G-means. The proposed method, with its impressive ascendancy, showcases enhancements of no less than 4.79% in classification accuracy, 7.26% in F1-Score, and an even more pronounced elevation of 5.27% in G-means. These consistent trends across two evaluation criteria emphasize the superior performance of the MIPF-LASSO method.

Overall, the MIPF-LASSO method achieves excellent results in feature selection, which not only helps improve the classification accuracy but also significantly enhances the G-mean. The observation of small standard deviations is an indicator of the MIPF-LASSO method’s consistent and stable performance. Therefore, the experimental results suggest that the method is not only effective in achieving high average values for performance metrics but also reliable and robust across different experimental conditions.

### 4.3. Ablation Experiment

To thoroughly assess the effectiveness of our innovative framework, a series of ablation experiments was executed to dissect the nuanced impact of variables within the model on the classification performance of the dataset. The experiments commenced with the application of a meticulously crafted feature selection framework, engineered to demonstrate a feature subset with wielding a profound influence. Subsequently, armed with these meticulously chosen feature subsets, the experiment delved into the classification tasks, meticulously considering an array of evaluation indicators, ranging from classical classification accuracy to nuanced metrics like G-mean and F1-Score, orchestrating a symphony of assessments to holistically evaluate performance. This methodical process endowed us with a profound understanding of the intricate influence wielded by the selected feature subset on the model’s overarching generalization performance.

**Figure 3:**
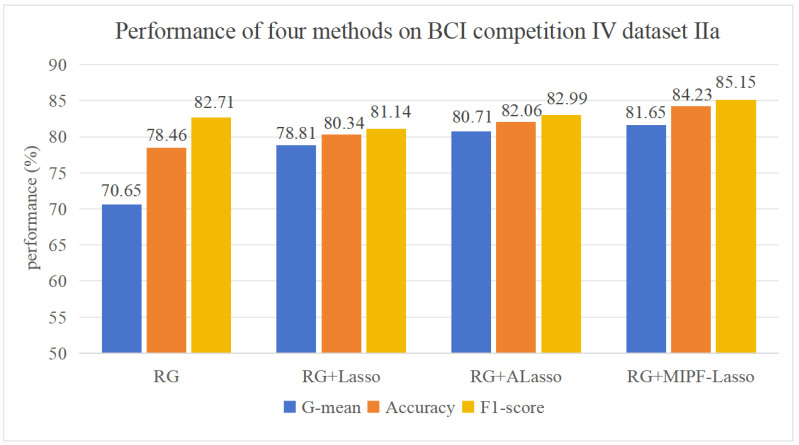
Results of ablation experiments on dataset IIa.

**Figure 4:**
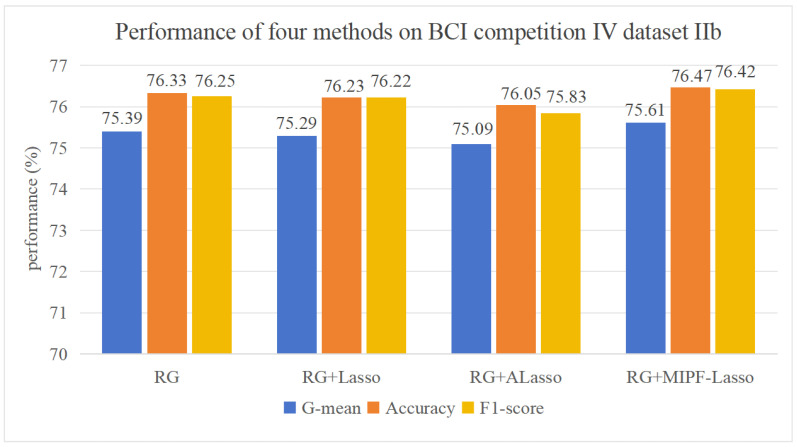
Results of ablation experiments on dataset IIb.

The interaction information, adaptive L1 penalization, and L1 penalization are selectively disabled from MIPF-LASSO in a meticulous sequence, birthing three distinct methodologies: Riemannian Adaptive LASSO, Riemannian LASSO, and Riemannian. The same meticulously experimental settings ensure an equitable stage for our comparative experiments in datasets IIa and IIb, respectively. The captivating results shown in Figure 3 and Figure 4, depicting the average classification performance, bear witness to the ascendancy of the RG MIPF LASSO method with the loftiest classification accuracy, G-mean and F1-Score.

In Figure 3, the classification accuracy, F1-Score and G-mean increase with the variable L1 penalty, adaptive L1 penalty, and interaction information one by one. Delving deeper, it becomes evident that the proposed method exhibits superior performance and surpasses alternative methods by a noteworthy margin. Specifically, the sequential addition of the three construct parameters manifests a remarkable improvement in classification accuracy, soaring from the initial 78.46% to the zenith of 84.23%. The F1-Score value changes from 82.71% to 85.15%. Concurrently, the G-mean experiences a meteoric rise, ascending from 70.65% to a pinnacle of 81.65%. This analysis underscores the tangible influence of the model’s variables on feature selection, thereby elevating the accuracy of the classification. Therefore, the proposed method showcases exceptional classification performance on dataset IIa.

Based on dataset IIb, the influence in classification accuracy and G-mean after adding LASSO and adaptive LASSO to feature selection in the Riemannian geometric framework is shown in Figure 4. The classification accuracy and G-mean of RG LASSO and RG adaptive LASSO were both found in the lower echelons compared to RG. This consequential outcome is attributed to the assumption inherent in adaptive LASSO and LASSO; the assumption leads the feature selection to potentially leave some relevant features out of the consideration [53]. The complex EEG data, harboring a non-linear relationship among features, disrupt this assumption and cast a discernible impact on feature selection. However, the interactive information is increased into the adaptive LASSO method, and the accuracy, F1-Score, and G-mean soar to 76.47%, 76.42%, and 75.61%, respectively, reaching the apogees in Figure 4. This indirectly indicates the importance of mutual information for feature selection. Furthermore, compared to the corresponding metrics of other methods as illustrated in Figure 4, the accuracy, F1-Score, and G-mean achieved by the proposed method are elevated by 0.14%, 0.17%, and 0.22%, respectively. This substantiates the efficacy and enhanced capabilities of feature classification for the proposed method in comparison to the other methods.

**Figure 5:**
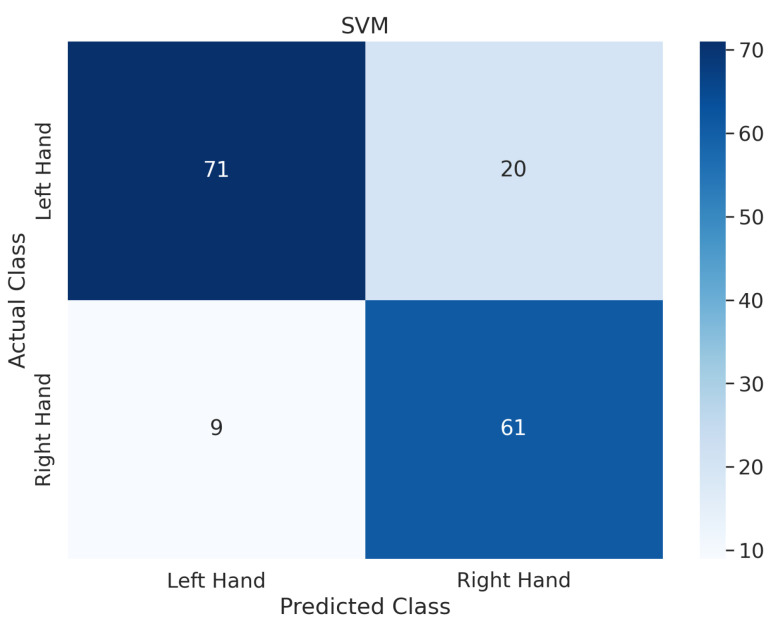
The confusion matrix of the proposed model for subject 1 on dataset IIa.

In addition to the above experiments, taking subject 1 from dataset IIa as an example, the confusion matrix shown in Figure 5 is calculated from the classifier output, where the rows represent the true classes and the columns represent the predicted classes by the SVM classifier, indicating that the proposed methods significantly improve the classification performance of each class.

In a concise summary of our experimental investigation utilizing the datasets IIa and IIb, the classification accuracy and G-mean achieved by the comparison methods are vividly illustrated in Figure 3 and Figure 4. Remarkably, through the incorporation of interactive information as a feature importance measure within the MIPF-LASSO model, the proposed MIPF-LASSO algorithm demonstrates a substantial enhancement in classification accuracy. This pivotal integration not only elevates its own performance but also establishes its superiority over other competing methods, affirming its effectiveness in the context of our experimental study.

## 5. Conclusions

In this paper, we propose a novel feature learning model, MIPF-LASSO, for effectively analyzing MI-EEG signals. To improve the classification performance, we fused the interaction information between features into the penalty term. Then, we introduced an adaptive L1 penalty and a weighted fusion penalty into the LASSO model to select the most valuable features. On the one hand, we developed an adaptive weight construction strategy using mutual information to evaluate the importance of features. By multiplying the regression coefficient corresponding to each variable by the adaptive weight, we were able to impose a differential penalty on each feature. On the other hand, based on the correlation information in the data, we introduced correlation-driven weights to penalize pairwise-fused differences between the coefficients and performed group selection of features. Furthermore, the coordinate descent algorithm was introduced to implement the proposed MIPF-LASSO method numerically. Experimental results on benchmark instances indicate that the proposed model is more suitable for classification and feature selection than existing models. Although our proposed model focuses on solving binary classification problems, it can also be applicable to multiple classification problems.

In the future, we will deepen and improve the MIPF-LASSO optimization scheme by more fully considering the relationships between features, making it more flexible and applicable to a variety of multiclassification scenarios. Further, apart from the time-domain information of EEG signals, it is also potentially valuable to consider the use of the information contained in the power features of some frequency bands for the recognition of MI-EEG signals, which helps to improve the classification performance [54]. We can explore incorporating a spectral feature extractor into the MIPF-LASSO model.

## Figures and Tables

**Table 1:** Performance comparison between MIPF-LASSO and baselines on dataset IIa.

	Methods	Accuracy (%)	G-Mean (%)	F1-Score (%)
		**(Mean ± std)**	**(Mean ± std)**	**(Mean ± std)**
Traditional method	FBCSP + SVM [48]	75.93 ± 14.76	72.69 ± 20.37	74.49 ± 18.47
	FBCSP + LDA [49]	73.75 ± 14.28	70.73 ± 18.00	75.72 ± 12.89
Deep learning method	SpectralSpatialCNN [50]	76.84 ± 13.63	74.58 ± 15.44	76.95 ± 15.28
	DeepConvNet [51]	64.34 ± 17.89	61.79 ± 19.90	60.17 ± 22.70
	EEGNet [52]	65.68 ± 18.22	55.94 ± 29.14	64.18 ± 25.59
	MIN2Net [47]	65.46 ± 15.60	64.13 ± 16.60	64.54 ± 18.35
Proposed method	MIPF-LASSO + SVM	84.23 ± 13.44	81.65 ± 19.05	85.15 ± 10.93

**Table 2:** Performance comparison between MIPF-LASSO and baselines on dataset IIb.

	Methods	Accuracy (%)	G-Mean (%)	F1-Score (%)
		**(Mean ± std)**	**(Mean ± std)**	**(Mean ± std)**
Traditional method	FBCSP + SVM [48]	69.25 ± 12.32	67.39 ± 13.66	68.01 ± 12.98
	FBCSP + LDA [49]	67.95 ± 12.22	67.17 ± 12.76	67.90 ± 12.63
Deep learning method	SpectralSpatialCNN [50]	71.68 ± 13.27	70.34 ± 14.03	69.16 ± 14.88
	DeepConvNet [51]	61.44 ± 16.23	59.76 ± 17.17	59.85 ± 18.44
	EEGNet [52]	66.34 ± 15.83	62.23 ± 21.23	65.12 ± 21.32
	MIN2Net [47]	60.06 ± 14.23	58.42 ± 15.17	59.87 ± 15.82
Proposed method	MIPF-LASSO + SVM	76.47 ± 13.86	75.61 ± 14.13	76.42 ± 13.69

## Data Availability

Data will be made available on request.
